# Long-term results of 32-mm alumina-on-alumina THA for avascular necrosis of the femoral head

**DOI:** 10.1007/s10195-011-0174-7

**Published:** 2012-01-17

**Authors:** Giuseppe Solarino, Andrea Piazzolla, Angela Notarnicola, Lorenzo Moretti, Silvio Tafuri, Silvana De Giorgi, Biagio Moretti

**Affiliations:** 1 Department of Neuroscience and Sense Organs, Orthopaedic and Traumatology Unit, Faculty of Medicine and Surgery, General Hospital, University of Bari, Piazza Giulio Cesare 11, 70124 Bari, Italy; 2 Department of Biomedical Sciences-Hygiene Section, Faculty of Medicine and Surgery, General Hospital, University of Bari, Bari, Italy

**Keywords:** Avascular necrosis, Hip arthroplasty, Alumina-on-alumina ceramic bearings

## Abstract

**Background:**

Ceramic bearings in total hip arthroplasty (THA) have been introduced in clinical practice to minimize the problem of polyethylene particle–induced osteolysis. The aim of the study is to report the results of 68 consecutive alumina-on-alumina THAs done in 61 patients for avascular necrosis (AVN) of the femoral head.

**Materials and methods:**

In all implants a press-fit cup was used; it was combined with a 32-mm alumina head and with titanium-alloy stems. The mean age at surgery was 50 years. At an average follow-up of 13 years two hips have been revised, one for periprosthetic infection and one for excessive abduction of the cup.

**Results:**

No revision for aseptic loosening is recorded; one anatomical cementless femoral stem had radiological evidence of definite aseptic loosening. No dislocations occurred, and no osteolysis was observed.

**Conclusions:**

The results support the application of alumina-alumina THA for long-lasting replacements.

## Introduction

Avascular necrosis (AVN) of the femoral head is due to a compromised blood supply to the femoral head, leading to necrosis of osteocytes, localized resorption and collapse of bone. The management of femoral head osteonecrosis remains a therapeutic dilemma, and also the American Association of Hip and Knee Surgeons does not support any standardized protocol [1]. Various procedures have been advocated to avoid the need for total hip replacement. Pulsed electromagnetic field treatment may be indicated in the early stages of osteonecrosis to either preserve the hip or delay the time until surgery. In a retrospective study, Massari et al. [2] showed that pulsed electromagnetic fields preserved 94% of Ficat stage I or II hips. Pain disappeared after 60 days of stimulation in 53% of the patients and was of moderate intensity in 26%. The need for total hip arthroplasty was significantly higher in patients with Ficat stage III disease than in patients with Ficat stage I or II. It was concluded that pulsed electromagnetic fields are able to stimulate the articular cartilage in relation to the catabolic effect of inflammation and subchondral bone marrow edema, and to promote osteogenic activity at the necrotic area to prevent trabecular fracture and subchondral bone collapse. In the pre-collapse stage, core decompression combined with and bone grafting should be chosen: Wei and Ge [3] reported a survivorship of 81% in a series of 162 patients with excellent results in 93.3% of cases in stage II and good results in 87% of the patients in stage III of Association Research Circulation Osseous. For late stages of the disease, THA, providing early pain relief and good functional outcome, is claimed to be the treatment of choice; however, some studies have reported high mechanical failure rates, indicating that osteonecrosis itself could represent a risk factor [4–6]. Osteolysis, as a result of polyethylene wear debris, remains one of the most important failure mechanisms in THA, and alternative bearing surfaces have been introduced in clinical practice to minimize the negative effects of wear on implant survival. As a result, there has been a renewal of interest in hard bearing surfaces for total joint arthroplasty, including both metal-on-metal and ceramic-on-ceramic components. However, despite excellent tribological (lubrication, friction, wear) properties, complications should be considered even with these materials, because metallic or ceramic debris may be produced; in addition, the biologic response to debris generated from alternate bearings has not been fully elucidated. Metal-on-metal bearings raise associated issues of metal hypersensitivity, toxicity, an increase in blood and urine metal ion levels, capsular lymphocytic aggregation and growth of pseudo tumor, which can lead to neuropathy; for ceramic-on-ceramic bearing surfaces, issues of ceramic quality, precise manufacture and contact surface geometry, including optimal clearance, the possibility of a transient squeaking sound and brittle fracture must be considered [7].

The purpose of this retrospective study was to report the results of a series of 68 consecutive alumina-on-alumina THAs done for AVN in 61 patients.

## Materials and methods

This clinical retrospective study included patients who were undergoing THA for AVN of the femoral head from January 1995 to December 1998 at our Operative Unit of Orthopaedics and Traumatology. The study was authorized by the Local Ethics Committee and was performed in accordance with the ethical standards of the 1964 Declaration of Helsinki. We investigated 68 THAs performed in 61 consecutive patients.

All of the patients received information about the aims of the study and signed an informed consent form prior to undergoing the clinical and diagnostic tests that were included in the study protocol. Continuous variable were expressed as mean, standard deviation and range. Categorical variables were presented as percentages, with 95% confidence intervals (CI). The *t* test for paired samples was carried out to compare the Harris Hip Scale score evaluated before the operation and at the most recent follow-up.

The series was composed of 35 men and 26 women; the average age of the patients at the time of the operation was 49.9 ± 10.1 years (range 29–72). Diagnosis of osteonecrosis was made on an anteroposterior view of the pelvis and a lateral radiograph of the involved hip. The disease stage was graded according to the system of Ficat and Arlet [8]: 35 patients presented with stage III and 33 patients with stage IV. In all cases magnetic resonance images were obtained: according to Steinberg’s classification [9] 12 patients had stage III, 25 patients stage IV, 18 patients stage V and 13 patients stage VI. In all patients, the diagnosis was confirmed by pathological examination of the resected femoral head. Operations, involving the right side in 37 cases and the left in 31 were performed in a conventional turbulent flow theatre using the direct lateral approach described by Hardinge [10].

The press-fit cup, hammered into a 2-mm underreamed acetabulum, consisted of a pure titanium core with a titanium alloy mesh: its shape is grossly hemispherical (polar flattening and circumferential gutters, Triradius-M cup), with one hole on the apex for the liner, inserted by a conical sleeving; it was always combined with a 32-mm femoral head. Both the inlay and the head were made of dense polycrystalline surgical-grade alumina (Al_2_O_3_).

**Fig. 1:**
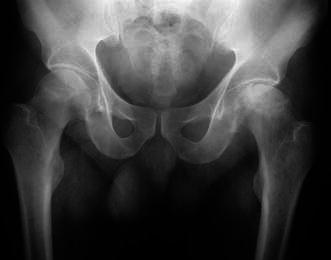
Preoperative radiograph: bilateral avascular necrosis of the femoral head

**Fig. 2:**
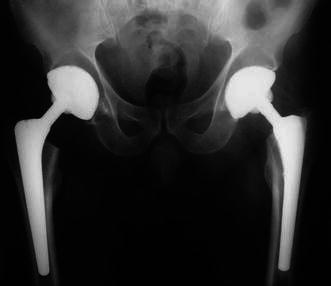
Radiograph of bilateral THA at follow-up: anatomic stem on the *left* side and right straight stem on the *right* side

Two additional fixation screws were used in three cases, in the two additional holes of the shell. The alumina head was anchored via a Morse taper on three different femoral components made of anodized titanium alloy (TiAl_6_V_4_): one smooth and collared cemented stem in 14 patients (20.6%; 95% CI = 10.9–30.2) and two collarless cementless stems. One was anatomical and smooth with a trochanteric wing and porous coating mesh medially in the proximal part and was used in 26 cases (38.2%; 95% CI = 26.7–49.8); one was straight, with a three-dimensionally tapered wedge with anti-rotational ribs in the proximal part and a rough blasted surface, and was used in 28 cases (41.2%; 95% CI = 29.5–52.9) (Figs. 1, 2). All the components were manufactured by Ceraver (Ceraver Osteal, Roissy, France).

In the hybrid implants, a distal cement restrictor was used, the medullary canal was cleaned with saline lavage, and an injection gun was employed together with digital pressurization of the cement. Perioperative care was the same for all patients: thromboembolic (heparin administration and compression stockings) and antibiotic prophylaxis; passive motion exercises with the assistance of a therapist immediately after the operation; patients were free to walk with two supports after 3 days for about 6 weeks and thereafter full weight-bearing was usually allowed. Clinical and radiographic follow-up was performed at 6 weeks, 3 months, 6 months and 1 year after the operation, and yearly thereafter. Serial anteroposterior radiographs of the pelvis were analyzed by the same observer, who had not been involved in the operations.

Harris hip ratings [11] were determined preoperatively and at each follow-up examination. A Harris Hip Scale score of 90 points or more was defined as an excellent outcome; 80–89 points, a good outcome; 70–79 points, a fair outcome; less than 70 points, a poor outcome. Preoperatively, the average was 37.2 points (minimum 16, maximum 63).

The inclination angle of the cup was measured as the angle formed by the inter-teardrop line and a line through the superior surface of the acetabular component; neutral cups were defined as those ranging from 35° to 49°, vertical as greater than 49° and horizontal as less than 35° [12]. In the X-rays taken at discharge, 48 cups were neutral (70.6%; 95% CI = 59.8–81.4), 13 vertical (19.1%; 95%; CI = 9.8–28.5), and 7 horizontal (10.3%; 95% CI = 3.1–17.5).

Linear wear of the acetabular component was determined by measuring the change in the shortest distance between the center of the femoral head and the periphery of the acetabular component as seen on the immediate postoperative radiograph compared with that seen on the radiograph made at the latest follow-up evaluation, as described by Livermore et al. [13]. Measurements were made with calipers that had an accuracy of 0.5 mm. Correction for magnification was performed on the basis of the radius of the femoral head.

According to Bizot [14] loosening of the socket was defined as cup migration exceeding 3 mm, angular rotation exceeding 3° or a continuous radiolucent line wider than 2 mm according to the zones described by DeLee and Charnley [15].

On the femoral side, parameters investigated included subsidence of the stem, calcar resorption and progression of radiolucent lines according to the seven zones described by Gruen [16]: loosening of the cemented stem was defined as migration exceeding 3 mm or a continuous radiolucent line wider than 2 mm; cementless components were classified as ingrown bone, fibrous stable or unstable, according to Engh’s criteria [17]. Heterotopic ossifications were graded according to the classification of Brooker [18].

One patient (two hips), the eldest of the group, died with both THAs well functioning at 10 years and 10 years and 5 months after the operations; three patients (three hips) were lost to final follow-up; a 36-year-old lady was known to have had her right THA revised elsewhere because of excessive painful abduction of the cup; one patient had revision of all components as a result of sepsis at 6 years and 8 months postoperatively.

A survivorship analysis according to the Kaplan-Meier method [19] was performed with revision for any reason as one end point and with infection at the time of follow-up as the other end point. The survival curve was derived from the cumulative survival rate over time. The standard error, given as a percentage, and the 95% confidence intervals were calculated.

## Results

At an average follow-up of 155 ± 19.9 months (range 132–180 months), 61 hips in 55 patients were available for clinical and radiological examination and were the subject of this retrospective study. Before surgery the Harris Hip Scale score was 37.2 ± 10.5 points (range 16–63), while at the follow-up it was 90.7 ± 5.8 points (range 68–100; *t* = −38.2; *P* < 0.0001): 52 were considered excellent, 7 good and 2 fair.

None of the cups showed evidence of migration, angular rotation or radiolucent lines: all of them were thus considered well fixed and stable with bone-ingrowth. A revision was only needed for a vertical cup. When we compared the vertical, neutral and horizontal cup groups, we did not find any differences in the survival rate or complication rate (chi-square = 0.42; *P* = 0.8). None of the cemented stems had migrated or tilted, but three of them had a non-progressive radiolucent line of <1 mm at the cement-bone interface in zones 1 and 7. The radiological exam did not indicate any sign of osteolysis on either the femoral or acetabular sides, and wear measurement was undetectable.

Four (15.4%; 95% CI = 1.5–29.2) of the 26 anatomical cementless femoral stems were considered unstable, and one of these had evidence of definite aseptic loosening. Of the remaining 22, 9 (34.6%; 95% CI = 16.3–52.9) were considered fibrous-stable with pedestal formation and cancellation of the femoral calcar (zone 7), and 13 (50%; 95% CI 30.8–69.2) showed bone ingrowth. Although there seems to be a discrepancy between the excellent results of the cups and the good/fair results of the anatomical cemented stems, no correlation with ceramic insert wear was found, but rather with the poor design of the stem of the femoral component. This was so much the case that the anatomical stem was substituted with a straight stem by the producer (Ceraver, Ceraver Osteal, Roissy, France). The three-dimensionally tapered wedge with anti-rotational ribs in the proximal part and a rough blasted surface showed excellent performance also when they were investigated by instrumental exams [20]. All of the 28 cementless straight stems were considered stable, but 11 of these showed thinning of the calcar femorale (zone 7); pedestal formation was never noticed. Heterotopic ossifications were observed in 16 (26.2%; 95% CI = 15.2–37.3) of the 61 hips: among these, 10 (62.5%; 95% CI = 38.8–86.2) were classified as Brooker class I, 5 (31.2%; 8.5–53.9) as Brooker class II and 1 hip (6.2%; 95% CI = −5.5–18.1) as Brooker class III. None was classified as Brooker IV. None of the hips were dislocated, none of the ceramic components (femoral head or acetabular inlay) broke out, and osteolysis was undetectable.

**Fig. 3:**
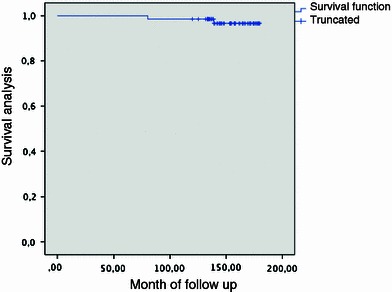
Kaplan-Meier analysis shows >95% of overall survival in patients treated with alumina-on-alumina THAs for AVN of the femoral head

**Fig. 4:**
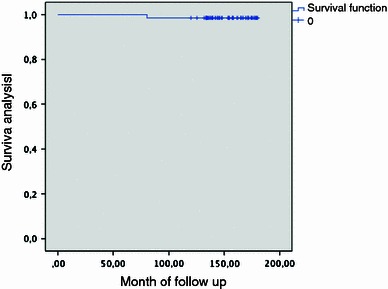
Kaplan-Meier analysis shows >95% of infection-free survival in the patients included in the study

Kaplan-Meier survivorship curves showed >95% patient survival at 180 months (estimate = 177.8; standard error = 1.64) (Fig. 3). Infection accounted for <5% of revised patients (estimate = 178.6; standard error = 1.47) (Fig. 4).

## Discussion

This study demonstrates that the use of alumina–alumina THA for AVN of the femoral head was successful at an average follow-up of 13 years. AVN is due to compromised blood supply to the femoral head that leads to resorption and collapse of the bone; the treatment of choice in late stages of this disease is hip replacement because of the high likelihood of providing early pain relief and good functional outcome. In the attempt to replace only the pathological cartilage and bone, femoral surface hemiarthroplasty has been indicated as a potential strategy, but the clinical outcome with such procedures has been controversial: its rate of failure was reported to be 27% at short-term follow-up if conversion to a THA was the endpoint, and up to 65% if revision and/or poor Harris Hip Scale score was considered as failure [21]. On the contrary, using the modern modified surgical approach of resurfacing arthroplasty, it is possible to obtain good results [22].

Even if excellent results with conventional bearings are claimed [23], usually when a THA is performed as the primary procedure for AVN, problems with polyethylene wear can arise in the medium and long term: in fact several studies have demonstrated high rates of failure with either cemented or cementless components. Alternative bearing surfaces, other than polyethylene, have been introduced in clinical practice to minimize the negative effects of wear on hip arthroplasty survival; the new generation all-ceramic and all-metal prostheses have demonstrated, both clinically and in the laboratory, low friction and low wear that theoretically should result in less particulate debris.

Alumina-on-alumina THAs have functioned well in vivo for more than 12 years with remarkably low wear even when first generation implants have been used: Jazrawi et al. [24] reviewed 60 hips replaced in young patients at a minimum follow-up of 10 years and reported an annual mean wear rate of the alumina bearings of 0.016 mm despite unsatisfactory results of the cementless components. Garcia-Cimbrelo et al. [25] analyzed 83 ceramic-on-ceramic Mittelmeier cementless THAs and, although 11 acetabular components and 7 stems had to be revised and radiographic signs of cup and stem loosening appeared respectively in 53% and in 15% of the hips, significant wear was not observed in any of the hips on plain radiographic films.

As supported by our clinical experience, in this case study of 68 arthroplasties we verified no wear of the ceramic at an average follow-up period of 13 years. Comparable results were verified by previous clinical studies [24, 25]. Neverthless, specific complication must be accounted for with hard materials: current metal-on-metal bearings are self-polishing and based on cobalt-chromium-molybdenum alloys with varying carbon contents. Debris from such bearings are responsible for increased metal ion levels in the blood and urine, capsular lymphocytic aggregation and delayed-type hypersensitivity. Eswaramoorthy et al. [26] described two cases of aseptic lymphocytic vasculitis with a lesion-type tissue response in four Metasul metal-on-metal THAs revised because of unexplained pain. Harvie et al. [27] reported 17 cases of a soft tissue mass presenting in patients with metal-on-metal resurfacing arthroplasty: two of them presented with femoral nerve palsy, and the intraoperative findings revealed a large, solid and fusiform pseudotumour mass comprising a firm pseudocapsule within the muscle and containing necrotic caseous material. In our case revised for a periprosthetic infection, we found no signs of synovitis or of metallosis at the 6-year follow-up.

Using with skin-patch tests, Park et al. [28] had already demonstrated a hypersensitivity reaction to cobalt in patients with early osteolysis after contemporary metal-on-metal THAs, and Savarino et al. [7] showed high levels of cobalt and chromium with metal-on-metal articulations and negligible serum metal ion contents in ceramic-on-ceramic THAs; they concluded that, due to the higher ion release, metal-on-metal coupling must be prudently considered, especially in young patients. Hart et al. [12], measuring the acetabular inclination angle and whole blood metal ion levels in patients with well-functioning Birmingham hip resurfacing, identified a threshold level of 50° cup abduction, above which the mean blood cobalt and chromium levels increased significantly. The excessive abduction of the cup is also a potential cause of a squeaking sound in ceramic-on-ceramic arthroplasties, as stated by Restrepo et al. [29], who reported an occurrence of 2.7% at their institution. Capello et al. [30] reported 0.8% transient noisy hips in a prospective, randomized, multicenter study of 475 THAs reviewed at an average 8-year follow-up.

No patients complained of any noises in the series of 319 ceramic-on-ceramic THAs performed by Garcia-Cimbrelo et al. [31], using alumina components manufactured by the same company as in our study. Ceramic materials have been recognized as increasingly important for their chemical and physical characteristics; in fact they present excellent biocompatibility, a low friction coefficient and high wear resistance, solving the complication of osteolysis secondary to polyethylene wear in young and active people such as patients with AVN.

In our case series, the radiological exam did not indicate any sign of osteolysis on either the femoral or acetabular sides, as supported in the literature by Ha et al. [32], who recorded neither pelvic nor femoral osteolysis at the 5–6-year follow-up on 78 THAs in which 28-mm alumina-on-alumina bearings had been used; when wear measurement was possible, it was undetectable.

A similar population as in our study was previously investigated in the literature utilizing identical components by the same producer. With the same ceramic components we used, i.e., Ceraver with a 32-mm femoral head diameter, several studies have been carried out by French authors on young patients affected by AVN at the medium and long term: on 41 patients operated on for femoral head osteonecrosis, ceramic wear was undetectable and osteolysis absent at a follow-up to 23 years, even with unsatisfactory performance of the plain alumina cup [33]. The results are comparable between this study and our case report.

These data confirmed the successful results of a previous report in which the implant survival rate after 9 years was 97.4% if revision of the prosthesis for aseptic loosening was considered the end point [14]. Later, Bizot et al. [34] reported an overall survival rate of 98.3% at a 6- to 11-year evaluation on 71 consecutive hybrid alumina-on-alumina THAs performed in patients aged 21–54 years and affected by AVN in 31% of the cases.

More recently, Kim et al. [35] investigated 93 cementless THAs using alumina-on-alumina ceramic bearings in 64 young patients with femoral head osteonecrosis using radiographs and computed tomographic scans. At the 10-year minimum follow-up, none of the hips had revision or aseptic loosening, and CT scans demonstrated no acetabular or femoral osteolysis [35]. At a longer follow-up, from 18.5 to 20.5 years, Hamadouche et al. [36] evaluated 118 hips: in the 106 (90%) patients available at the last follow-up, wear of the prosthetic components was undetectable on plain radiographs, and, when revision was needed, its mean acetabular rate was <0.025 mm/year; none of the patients had an infection, dislocation or fracture of the ceramic components. As in the latter report, in our series there were no dislocations: using a 32-mm femoral head, which is larger than the more popular 28 mm head, could explain the absence of this complication.

In our opinion consideration should be given when THAs are performed in patients affected by AVN, because this disease is considered to be a risk factor. The dislocation rate is expected to be much higher in osteonecrosis than in osteoarthritis patients according to the data from Ortiguera et al. [37] because patients with osteonecrosis have much less stiffness than patients with osteoarthritis and therefore might potentially obtain a higher range of motion that would, in turn, make them more susceptible to dislocation. Berry et al. [38] studied the cumulative risk for dislocation in a study of more than 6,600 hips and quantified a more than twofold greater risk in patients with osteonecrosis than in patients with osteoarthritis (14.1% compared with 6.4%); their study demonstrated that dislocation rates continue to increase with longer follow-up, perhaps reflecting the effects of neuromuscular deterioration and especially the wear at the metal-on-polyethylene articulating surface.

According to Millar et al. [39], even using uncemented ceramic-on-ceramic THAs in young adults, clinical scores were significantly better in patients affected by osteoarthritis than in patients affected by osteonecrosis at 6 months postoperatively, and the outcome was good to excellent for 85% of patients in the osteonecrosis group and 90% of patients in the osteoarthritis group.

The main limitation of the current study is the length of the enrollment period needed to obtain a sufficient number of patients, which is still quite small; however, contrarily, it might be a strength if one considers that we performed only an average of four ceramic-on-ceramic THAs per year in patients with AVN. Thus, this is an interesting sample for Orthopedic Departments in which small numbers of joint replacements are performed. In other words, not reporting major complications in our series with a minimum follow-up of 132 months, we believe that ceramic hip arthroplasty is a reliable procedure that should be recommended in young active patients with a long life expectancy, as patients with AVN usually are. In this population, wear is in fact usually higher. Although intracellular ceramic wear debris can be seen in the periarticular tissues, the ceramic particulate eventually causes a poorly aggressive inflammatory reaction, because it is tolerated much better by the organism than the one secondary to metal-on-metal articulation.

Such biologic inertness, the excellent tribologic properties, the extreme hardness and the good surface finish give ceramics excellent biocompatibility and allow using femoral heads larger than 28 mm, thus simultaneously providing a low friction coefficient, high wear resistance, an increased range of motion and a decreased dislocation rate. It must be remembered that the success of THAs in patients with AVN is connected with the technical difficulties while performing prosthetic surgery. The implant should be stable with correct muscle tension to avoid initial microseparation and eventually damage of the liner rim. The use of ceramics requires a precise surgical technique also paying attention to the small details. Furthermore, describing the technical difficulties of the surgical procedure should be useful as it provides support for general orthopedics practicing total hip replacement for AVN of the femoral head. This will lead to long-lasting total hip replacements.

On the basis of the results of this study, we support the use of ceramic-on-ceramic THAs for patients with AVN of the femoral head. This study confirms the improvements acquired with large-diameter (32 mm or more) ceramic heads with low incidence of complications at long-term follow-up.
